# A trajectory similarity computation method based on GAT-based transformer and CNN model

**DOI:** 10.1038/s41598-024-67256-7

**Published:** 2024-07-13

**Authors:** Liu Dongjiang, Li Leixiao, Li Jie

**Affiliations:** College of Data Science and Application, Inner Mongolia University of Technology, Huhhot, 010080 China

**Keywords:** Information technology, Scientific data

## Abstract

Trajectory similarity computation is very important for trajectory data mining. It is applied into many trajectory mining tasks, including trajectory clustering, trajectory classification and trajectory search etc. So efficient trajectory similarity computation method is very useful for improving trajectory mining result. Nowadays many trajectory similarity computation methods have been proposed. But most of them can not be applied into long trajectories similarity calculation efficiently. So a new algorithm called TrajGAT is proposed. This algorithm can calculate similarity for long trajectories. It treats long trajectory as a long sequence. By doing so, long-term dependency of long trajectory is considered by this algorithm while computing similarity value. But, the spatial feature of long trajectories is not considered. As long trajectory can be presented in many different shapes, if two long trajectories are judged as similar trajectories, the outline shape of these two trajectories should be similar as well. To solve this problem, a new trajectory similarity computation method is proposed in this paper. This method not only takes the long-term dependence feature into consideration, but also considers the outline feature of long trajectory. The proposed method employs GAT-based transformer to extract long-term dependence feature from long trajectory. And it applies Convolutional Neural Network to extract outline feature.

## Introduction

With the rapid development of GPS devices and network technology, trajectory data collection becomes easier. As trajectory data are created by moving objects, such as people, vehicles and animals etc, the behaviors and habits of these moving objects can be researched based on trajectory data. A trajectory is composed of a series of triplets. Each triplet contains a geospatial coordinate set and a timestamp. The triplet can be presented as $p=(x, y, t)$. *x* is longitude value of location *p*. *y* is latitude value of location *p*. *t* is a timestamp at which geospatial coordinate set of location *p* is collected. So a trajectory can be represented as a set $T=\{p_0, p_1, ..., p_n\}$. As trajectory dataset contains great amount of information, many trajectory mining algorithms are proposed. These algorithms can be classified into different categories, including periodical pattern mining algorithms^1,2^, sequential pattern mining algorithm^3,4^, moving together pattern mining algorithm^5,6^, trajectory classification algorithms^7–9^ and trajectory clustering algorithms^10–12^ etc. In all these trajectory mining algorithms, trajectory similarity computation algorithms are widely used. Thus a well-performed trajectory similarity computation method is very important for improving the trajectory mining result. So it is worth making a profound research about trajectory similarity computation.

Nowadays many trajectory similarity computation methods are proposed. These methods can be classified into two categories. The first one is non-learning based methods. The second one is learning based methods. Algorithms of the first category try to calculate the distance of trajectories directly. This kind of methods treats all the trajectory as spatial curve and tries to calculate the distance by using computational geometry. The typical calculation methods of this category contain Hausdorff^13^, DTW^14^, PDTW^15^, FastDTW^16^, LCSS^17^, LCSS based algorithm^18^, EDR^19^, ERP^20^, TWED^21^, EDwP^22^ etc. As each of these similarity calculation methods is proposed for a specific object, it is hard for a method to be applied to other objects. To overcome the shortage, algorithms of the second category are proposed. These algorithms try to calculate vector for every trajectory. So each vector contains characteristics of a specific trajectory. Then the distance of different trajectories can be calculated based on these vectors. As embedding based methods are very popular in recent years, many related algorithms are proposed, such as ST2Vec^23^, TrajCross^24^, t2vec^25^, play2vec^26^, Traj2SimVec^27^, At2vec^28^, T3S^29^, NEUTRAJ^30^, TS-Join^31^ etc. Generally, the algorithms mentioned above are mainly proposed for short trajectory. As long trajectory contains more location points than short trajectory, performance is poor while applying these algorithms into long trajectory similarity calculation. Accordingly, Yao et al.^32^ proposed a long trajectory similarity computation method which is called TrajGAT. This algorithm treats long trajectory as long sequence and applies transformer to capture long-term dependence feature of long trajectory.

**Figure 1:**
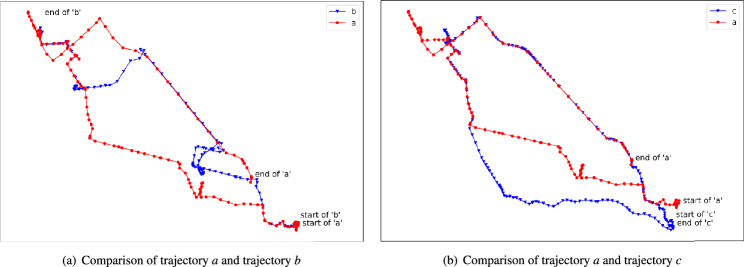
Comparison of trajectory *a*, trajectory *b* and trajectory *c*.

Even though TrajGAT algorithm is better than other algorithms in long trajectory similarity computation, it still needs to be improved further. TrajGAT algorithm tries to model a long trajectory by constructing a graph. Then the graph is input into a GAT-based Transformer. By using this GAT-based Transformer, a vector will be calculated for every trajectory. During the procedure, the area that contains location points of all the trajectories is separated into small regions. In this situation, if location points of two trajectories are very close, they will be put into same small region. As graph is constructed based on corresponding small regions, if most location points of two trajectories are put into same small regions, the vector of these two trajectories will be very similar. In this situation, these two trajectories can be judged as similar trajectories by TrajGAT algorithm. According to this, if two trajectories are very close to each other, they will be judged as similar trajectories by TrajGAT algorithm. But, as the outline of long trajectory can be different shapes, even though two trajectories are close to each other, they still can’t be similar trajectories. Because outline shape of two trajectories may be different. For example, in Fig. 1, obviously, the overlapping location point number of trajectory *a* and *b* is greater than the overlapping part of trajectory *a* and *c*. So more location points contained in *a* and *b* will be put into same small regions. Based on TrajGAT algorithm, trajectory *a* and *b* are more similar than *a* and *c*. But, the truth is that trajectory *a* and *c* are more similar. We can find that, not only many location points of *a* and *c* are overlapped, but also the outline shape of trajectory *a* and *c* are very similar. The outline shapes of both *a* and *c* are very close to a circle. But trajectory *b* is a unidirectional trajectory. So, while we try to find similar trajectories for a specific trajectory, the outline shape should also be considered.

In this situation, a new trajectory similarity calculation algorithm is proposed. This algorithm adopts GAT-based Transformer to extract long-term dependence feature from long trajectory. At the same time, to extract the outline feature of long trajectory, Convolutional Neural Network (CNN) is employed by the proposed algorithm. During the procedure, a matrix will be constructed for every long trajectory. Then CNN model is used to extract outline feature of long trajectory based on the corresponding matrix. Accordingly, the contribution of this paper is as below:

1. A new trajectory outline feature extraction method is proposed. This method is based on CNN model. By using this method, a vector that contains outline feature is generated for every trajectory.
2. A new trajectory similarity calculation algorithm is proposed. In this algorithm, not only the long-term dependence feature of long trajectory but also the outline feature of long trajectory are considered.

Rest of this paper is organized as follows. The proposed algorithm is described in section 2. Experimental results and discussion are presented in section 3. Section 4 concludes the contents of this paper and discusses future work related to trajectory similarity calculation.

## TrajBOAL algorithm

### Long-term dependence feature extraction

The proposed algorithm which is called TrajBOAL tries to construct a vector for each trajectory. Similarity value of two trajectories is calculated based on vectors. As location points of a long trajectory are interrelated, while constructing vector for every long trajectory, the long-term dependency should be considered. TrajGAT^32^ algorithm is proposed for calculating similarity value for two long trajectories. Long-term dependence feature of long trajectory is fully considered by this algorithm. So, the long-term dependence extraction method described in TrajGAT algorithm will be adopted by the proposed algorithm. Four steps are contained in TrajGAT algorithm while constructing vector for trajectory. Firstly, a hierarchical structure is constructed by using PR quadtree^33^. Then, generate a graph for every long trajectory based on constructed PR quadtree. Thirdly, a graph-based transformer layer is used to generate an embedding for a trajectory. Finally, TrajGAT algorithm applies deep metric learning framework to optimize the model parameters.

**Figure 2:**
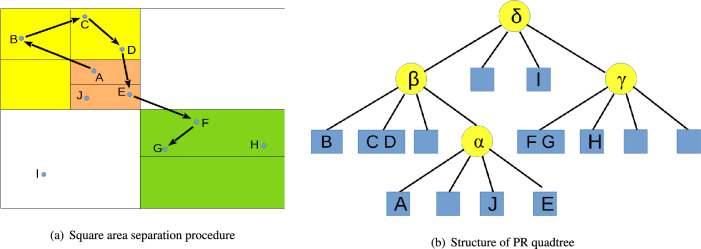
PR quadtree construction procedure.

In the first step of TrajGAT algorithm, PR quadtree is used to model the hierarchical spatial structure. During the procedure, the square area that contains all the trajectories will be separated into four square sub-areas. Then, the location point number contained in each area should be counted. If location point number contained in one sub-area exceeds a specific threshold, this sub-area should be separated into four square sub-areas further. This procedure will be continued, until the location point number of each area is no more than the defined threshold. The separation result is presented in (a) of Fig. 2.

While the separation process is finished, a PR quadtree can be constructed based on the separation result. The structure of PR quadtree is presented in (b) of Fig. 2. In this PR quadtree, every non-leaf node represents a square area, which is separated into four square sub-areas. And these four square sub-areas are represented by the four children of the non-leaf node. So, the root of this tree is corresponding to the square area that contains all the trajectories. After the PR quadtree is constructed, an embedding vector can be computed for every node of this PR quadtree. During the procedure, as PR quadtree can be treated as a graph, Node2Vec algorithm is employed to fulfil the embedding vector calculation task. Based on Node2Vec algorithm, every node of RP quadtree will get a vector. According to Fig. 2, location point *A* to *J* contained in the PR quadtree can be found in (a) directly. $\alpha$, $\beta$, $\delta$ and $\gamma$ are four areas. $\delta$ represents the whole square area that contains all the location points of trajectories. $\gamma$ is corresponding to the green square area. The yellow square area is represented by $\beta$. Obviously, the right bottom corner of yellow square area is covered by an orange square area. This area is corresponding to $\alpha$.

**Figure 3:**
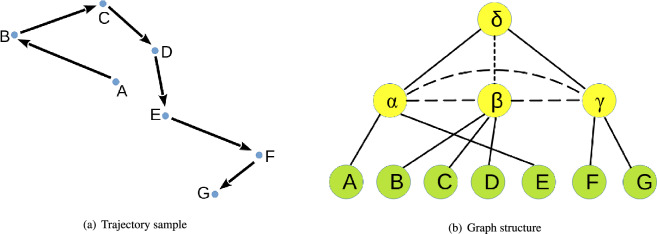
Constructing graph for a trajectory based on PR quadtree.

Then, an embedding vector should be constructed for every trajectory by using GAT-based transformer layer. Before that, a graph should be constructed for every trajectory. During the procedure, PR quadtree is used. Firstly, the square area that contains all the location points need to be found. Take the trajectory presented in (a) of Fig. 3 as an example. Location points *A* and *E* are contained in area $\alpha$. Points *B*, *C*, *D* and area $\alpha$ are contained in area $\beta$. Points *F*, *G* are contained in area $\gamma$. Area $\beta$ and $\gamma$ are contained in area $\delta$. So four areas are obtained from PR quadtree, which are $\alpha$, $\beta$, $\delta$ and $\gamma$. All the location point and all the square areas described above will be treated as nodes of the graph. Secondly, an edge set should be constructed. Two kinds of edges will be added into graph. The first kind is cross layer edge. If two nodes belong to different layers of PR quadtree, they will be connected by this kind of edge. The second type of edges is inner layer edge. If two nodes belong to same layer of PR quadtree, they will be connected by this kind of edge. The constructed graph is presented in (b) of Fig. 3.

While the graph is constructed, every node of this graph will get a vector. Then these vectors are input into GAT-based transformer layer. Vector of the $i-th$ node is calculated as follows.

1 $$\begin{aligned} v_{i}=\lambda _{i}+f_{i} \end{aligned}$$

$v_{i}$ represents vector of the $i-th$ node. Vector $\lambda _{i}$ represents the position encoding of the $i-th$ node. Vector $f_{i}$ represents feature of the $i-th$ node.

Before calculating vector $v_i$ for the $i-th$ node, feature vector $f_{i}$ of the $i-th$ node should be computed. Vector $f_{i}$ is calculated by concatenating three different vectors, which are $f^{l}_{i}$, $f^{r}_{i}$ and $f^{h}_{i}$. $f^{l}_{i}$ represents the coordinate feature vector of node *i*; $f^{r}_{i}$ represents the region feature vector of node *i*; And $f^{h}_{i}$ represents the hierarchical structure feature vector of node *i*. Calculation method of region feature vector $f^{l}_{i}$ is as follows.

2 $$\begin{aligned} \begin{aligned} x_i,y_i&=Normalize(lat_i,lon_i)\\ f^{l}_{i}&=MLP(x_i,y_i) \end{aligned} \end{aligned}$$

If node *i* is a location point, $lat_i$ and $lon_i$ represent the latitude value and longitude value of this point. If node *i* is a square area, $lat_i$ and $lon_i$ represent the latitude value and longitude value of center point of this square area. Function *Normalize*(.) represents min-max normalization method. Function *MLP*(.) represents Multi-layer Perceptron. Calculation method of region feature vector $f^{r}_{i}$ is as follows.

3 $$\begin{aligned} f^{r}_{i}=MLP(w_i,h_i) \end{aligned}$$

If node *i* is a location point, $w_i$ and $h_i$ are set to 0. If node *i* is a square area, $w_i$ and $h_i$ are set to the edge length value of corresponding square area. Hierarchical structure feature vector $f^{h}$ of each node can be obtained based on PR quadtree. As every node of PR quadtree has been assigned a vector by using Node2Vec algorithm, hierarchical structure feature vector of all the nodes can be obtained easily. For square areas $\alpha$, $\beta$, $\gamma$ and $\delta$, vector calculated based on Node2Vec algorithm will be treated as hierarchical structure feature vector directly. For all the location points, firstly, the leaf nodes of PR quadtree that contains them should be discovered. Then the vectors of these leaf nodes calculated by using Node2Vec algorithm need to be assigned to the corresponding location points as hierarchical feature vector. While every node getting a hierarchical structure feature vector, a special key should be added into these vectors.This special key is used to represent the hierarchical feature of all the nodes contained in PR quadtree. In this situation, the nodes that are close in space will share same special key. To obtain vector $f_i$ presented in Eq. (1), vector $f^l_i$, $f^r_i$ and $f^h_i$ will be concatenated. The procedure is as follows.

4 $$\begin{aligned} f_i=concat(f^l_i, f^r_i, f^h_i) \end{aligned}$$

**Figure 4:**
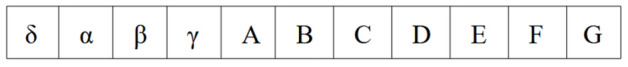
Structure of queue *L*.

To obtain vector $v_i$ presented in Eq. (1), a vector named $\lambda _i$ should also be calculated. It is a position encoding vector. The vector calculation procedure is executed based on the graph $T_g$ presented in (b) of Fig. 3. Vector $\lambda$ should be calculated for every node of graph $T_g$ by concatenating vector $\lambda ^s$ and vector $\lambda ^g$. Vector $\lambda ^s_i$ contains position information. Vector $\lambda ^g_i$ contains connection relationship of the nodes contained in graph $T_g$. While calculating vector $\lambda ^s_i$, sequential information of graph $T_g$ need to be modeled. During the modeling procedure, a queue *L* is constructed. The nodes contained in graph $T_g$ are input into queue *L* in order, from the first layer to the last layer. Structure of queue *L* is presented in Fig. 4.

Then dequeue operation is performed. While node *i* is obtained after performing dequeue operation, a vector $\lambda ^s_i$ should be constructed for this node. And this newly constructed vector is treated as the $i-th$ row of a matrix. While queue *L* becomes empty, a matrix is constructed. Each row of this matrix is corresponding to a specific node of graph $T_g$. The column number of this matrix is *d*/2. Sinusoidal value calculation method^34^ is used to calculate element values of vector $\lambda ^s_i$. Element value calculation method is as below.

5 $$ \lambda _{{ij}}  = \left\{ {\begin{array}{*{20}c}    {\sin (10000^{{\frac{j}{i}}} ),} & {{\text{if}}\;i\;{\text{is}}\;{\text{even}}}  \\    {\cos (10000^{{\frac{{j - 1}}{i}}} ),} & {{\text{if}}\;i\;{\text{is}}\;{\text{odd}}}  \\   \end{array} } \right. $$

where $\lambda _{ij}$ represents element value in row *i* and column *j* of the matrix. The value of row index *i* is contained in set [1, ..., *M*]. *M* is the number of nodes contained in graph $T_g$. As the length of vector $f_i$ is *d*, the value of column index *j* is contained in set $[1,...,\frac{d}{2}]$. $\lambda ^s_i$ is the $i-th$ row of the matrix. To model connection relationship of nodes, Laplacian position encoding^35^ is used. The method is presented below.

6 $$ \begin{aligned}   \Delta  &  = I - D^{{ - \frac{1}{2}}} AD^{{ - \frac{1}{2}}}   = U^{T} \Lambda U  \\  \lambda _{i}^{g} &  = MLP(top_{p} (U)) \\  \end{aligned}  $$

where *A* is adjacency matrix. *D* is degree matrix. $\Lambda$ contains all the eigenvalues. And *U* contains all the eigenvectors. $top_p(.)$ is an operation that slices the vectors corresponding to the *p* smallest non-trivial eigenvalues. $\lambda ^g_i$ is the Laplacian positional encoding result of node *i*. The length of vector $\lambda ^g_i$ is also $\frac{d}{2}$. Now vector $\lambda ^s_i$ and $\lambda ^g_i$ should be concatenated to form position encoding vector of node *i*. The procedure is as below.

7 $$\begin{aligned} \lambda _i =concat(\lambda ^s_i,\lambda ^g_i) \end{aligned}$$

Then the feature vector $f_i$ and the position encoding vector $\lambda _i$ can be summed up to get vector $v_i$, which is presented in Eq. (1). Vector $v_i$ contains the feature of node *i*.

After all the nodes of the constructed graph got a vector *v*, GAT-based transformer should be used to calculate a vector for the trajectory, which is corresponding to the graph. During the procedure, vectors of all the nodes are input into GAT-based transformer one by one. If the graph of a trajectory contains *M* nodes, output of GAT-based transformer will be a vector list, which is $\{h^P_1, h^P_2, ..., h^P_M\}$. Each vector $h^P$ is corresponding to a specific node of the graph. The embedding vector *e* of the trajectory is computed based on these vectors. Computation method is as below.

8 $$\begin{aligned} e=\sum ^{M}_{i=1}\frac{h^P_i}{M} \end{aligned}$$

As, while calculating vector $h^P_i$, vector $v_i$ is input into GAT-based transformer model at first, vector $v_i$ will be regarded as $h^0_i$. It is input into the first layer of GAT-based transformer model. The GAT-based transformer model contains *P* layers. Computation procedure of every GAT-based transformer layer is as follows.

9 $$\begin{aligned} \begin{aligned} h^{l+1}_i&=O^l_hconcat^H_{k=1}(\sum _{j\in N_i}w^{k,l}_{i,j}V^{k,l}h^l_j) \\ w^{k,l}_{i,j}&=softmax_j (\frac{Q^{k,l}h^l_i\cdot K^{k,l}h^l_j}{\sqrt{d_k}}) \end{aligned} \end{aligned}$$

where $N_i$ is a set that contains all the neighborhoods of the $i-th$ node. $Q^{k,l}$, $K^{k,l}$ and $V^{k,l}$ are $d_k\times d$ matrix. $O^l_h$ is a $d\times d$ matrix. *k* represents the number of attention heads, $k \in [1,...,H]$. $h^{l+1}_i$ is input into a Feed Forward Network (FFN). Residual connections and batch normalization module are contained in FFN. To calculate the parameter values of GAT-based transformer model, deep metric learning framework is employed by TrajGAT algorithm.

The procedure of long-term dependence feature extraction is presented in algorithm 1. Input value of this algorithm is a trajectory set. Output value is a vector set.

**Algorithm 1:**
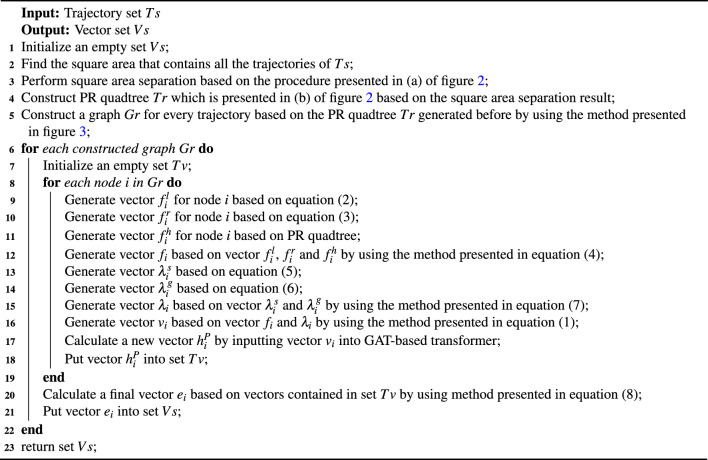
Long-term dependence feature extraction.

### Outline feature extraction

Long trajectory contains many location points. If these location points are connected based on the order of timestamps, the outline of long trajectory can be presented. We can find that the outline shape of long trajectory is very complicated. So, while calculating similarity value, the outline feature of long trajectory should be extracted. To fulfil the task, Convolutional Neural Network (CNN) is used. During the outline feature extraction procedure, two steps will be executed. In the first step, a matrix is constructed for every trajectory. In the second step, these matrices are input into CNN model one by one. Then a vector can be generated for every trajectory by CNN model. This vector contains outline information of the corresponding trajectory.

**Figure 5:**
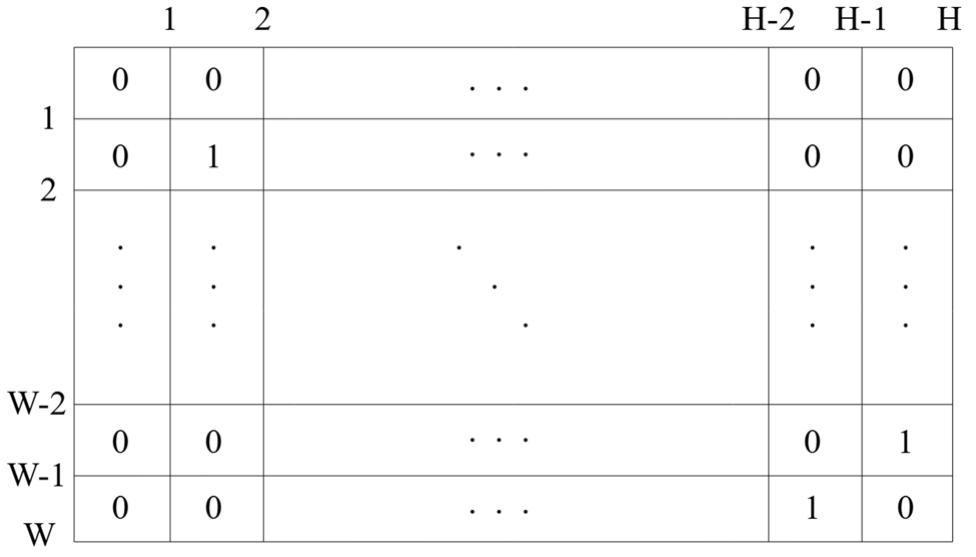
Constructed grids based square area which contains all the trajectories.

While constructing matrix for a long trajectory, Mercator projection method^36^ is used. Based on this method, geometric coordinate of a location point can be projected to Cartesian coordinate. While the projection task is fulfilled, two steps should be performed. Firstly, the square area that contains all the trajectories needs to be separated into rows and columns. During the procedure, the most important task is to identify the square area. To fulfil the task, latitude value and longitude value of all the location points will be collected. The minimum value and maximum value of latitude values and longitude values are extracted for setting the edges of the square area. Then the area between top and bottom horizontal edges are equally divided into *W* parts. The area between left and right vertical edges are equally divided into *H* parts. An example of square area division is presented in Fig. 5. As each row of the square area contains *H* grids and each column of the square area contains *W* grids, this square area can be treated as a $W \times H$ matrix. Element value of this matrix must be 1 or 0. Secondly, construct a $W \times H$ matrix for every trajectory. While constructing matrix $m_t$ for trajectory *t*, a row number and a column number should be computed for every location point of trajectory *t*. If the row number and column number of location point *p* are *r* and *c*, the element in the $r-th$ row and $c-th$ column of matrix $m_t$ will be set to 1. Obviously, many elements of matrix $m_t$ are not related to location points of trajectory *t*. Value of these elements will be set to 0. When matrix construction is finished, the outline feature of every trajectory can be extracted based on the corresponding matrix.

The calculation method of row number and column number is presented below.

10 $$\begin{aligned} \begin{aligned} w&=\lceil \frac{latitude-latatitude_{min}}{latitude_{max}-latitude_{min}}\cdot (W-1)\rceil +1 \\ h&=\lceil \frac{longitude-longitude_{min}}{longitude_{max}-longitude_{min}}\cdot (H-1)\rceil +1 \end{aligned} \end{aligned}$$

Suppose that the trajectory contains *N* different location points. Then $latitude_{min}=\min _{1\le n \le N} latitude_n$, $latitude_{max}=\max _{1\le n \le N} latitude_n$, $longitude_{min}=\min _{1\le n \le N} longitude_n$. $longitude_{max}=\max _{1\le n \le N} longitude_n$. $\lceil ... \rceil$ is ceil operation.

**Figure 6:**
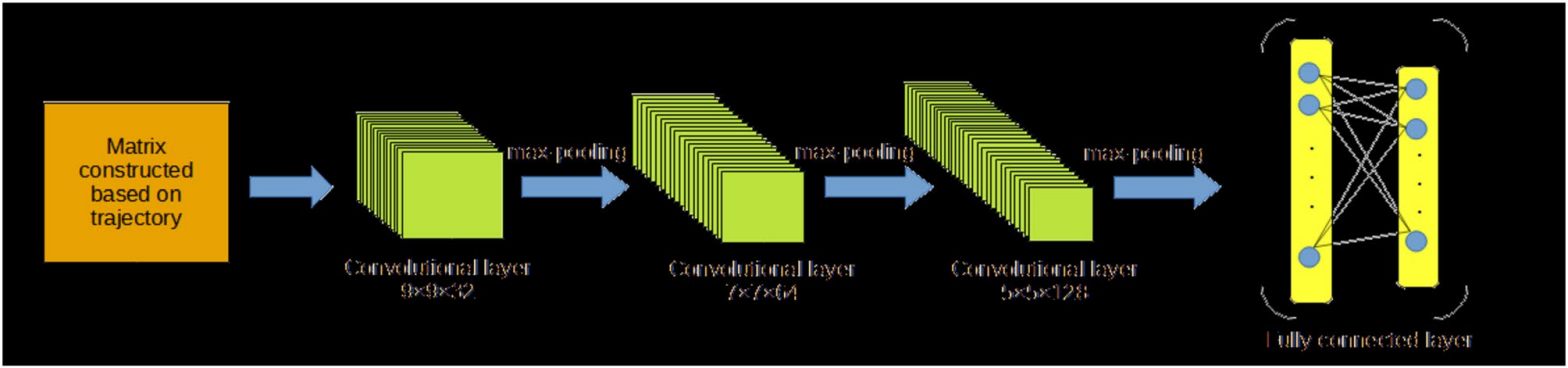
Structure of CNN model.

After projecting every trajectory into matrix, CNN model can be used to extract outline feature for every trajectory based on corresponding matrix. The CNN model used in the proposed algorithm contains three convolutional layers, three max-pooling layers and a fully connected layer. The structure of CNN model is presented in Fig. 6. The filter size of different convolutional layer are $9 \times 9$, $7 \times 7$ and $5 \times 5$. The filter numbers of different convolutional layers are 32, 64 and 128. Output value of the fully connected layer is a vector which contains 10 elements. This output vector contains outline feature of a trajectory.

**Figure 7:**
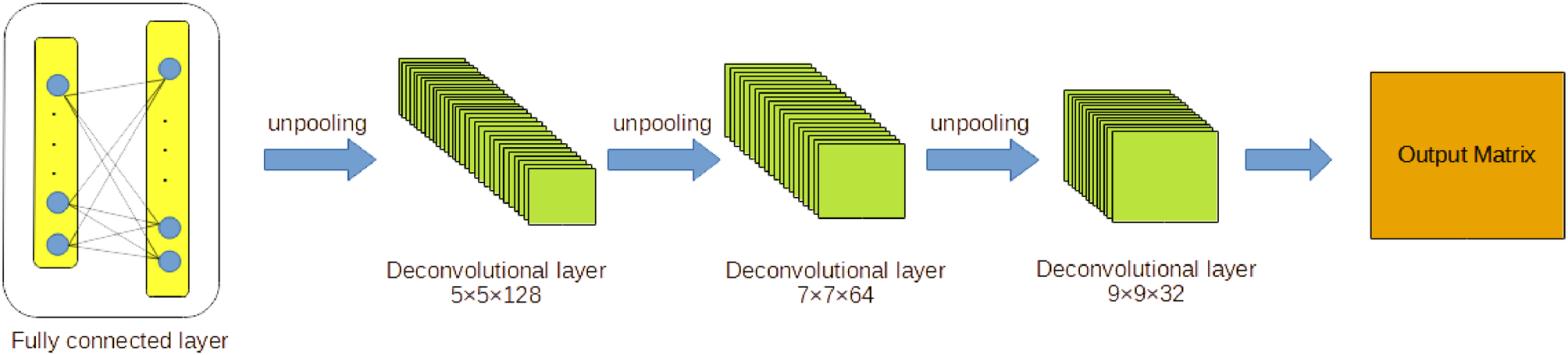
Decoder part of auto-coder neural network.

Initially, all the parameters of CNN model are generated randomly based on normal distribution. Auto-encoder is used to optimize the parameters of the CNN model. The CNN model described above is treated as encoder of the auto-encoder neural network. The output vector of CNN model will be treated as input value of decoder part of auto-encoder neural network. The structure of decoder part is presented in Fig. 7. The decoder part of auto-encoder neural network contains a fully connected layer, three deconvolutional layer and three unpooling layer. Fully connected layer of decoder part contains 512 neurons. Filter size of different deconvolutional layers are $5 \times 5$, $7 \times 7$ and $9 \times 9$. Filter numbers of different deconvolutional layers are 128, 64 and 32.

Suppose that the matrix which is input into CNN model is called $M_c$ and the output matrix of decoder part is called $M_o$. The loss function used to optimize parameters of auto-encoder should be as follows.

11 $$\begin{aligned} L=norm(M_c-M_o) \end{aligned}$$

Function *norm*(.) represents the normalization operation performed by using L-2 norm method. As matrix $M_o$ is obtained based on matrix $M_c$, these two matrices should be as close as possible. Thus minimum value of this loss function should be obtained while optimizing the parameters of auto-encoder neural network. The back-propagation algorithm is employed to perform parameter updating procedure and Adam optimizer is used to fulfil optimization task.

The procedure of outline feature extraction is presented in algorithm 2. Input value of this algorithm is a trajectory set. Output value of this algorithm is a vector set.

**Algorithm 2:**
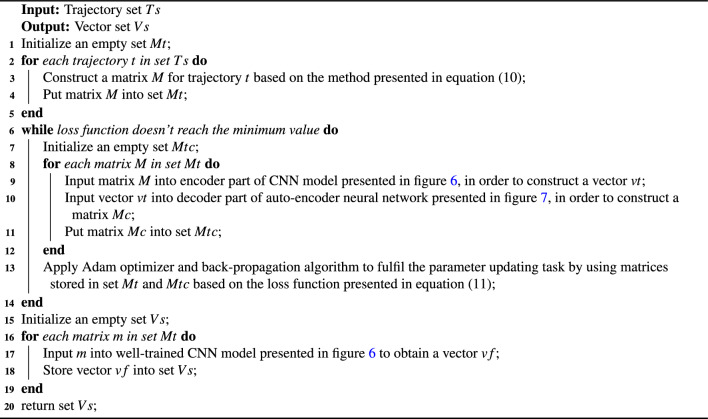
Outline feature extraction.

### Trajectory similarity calculation

After long-term dependence feature and outline feature of trajectory are extracted, the embedding vector can be constructed for every trajectory. Based on the embedding vectors of all the trajectories, similarity value of any two trajectories can be calculated.

Suppose that vector which contains the long-term dependence feature of the $i-th$ trajectory is $v_i$ and vector which contains the outline feature of the $i-th$ trajectory is $o_i$. The embedding vector $e_i$ of the $i-th$ trajectory can be constructed as follows.

12 $$\begin{aligned} e_i=concat(v_i,o_i) \end{aligned}$$

where function *concat*(.) represents concatenation operation of two vectors. While every trajectory gets its own embedding vector, similarity value of any two trajectories can be calculated by using cosine similarity. Calculation method is as below.

13 $$\begin{aligned} sim=\frac{e_i\cdot e_j}{\left| \left| e_i\right| \right| \left| \left| e_j\right| \right| } \end{aligned}$$

where $e_i$ is embedding vector of the $i-th$ trajectory, $e_j$ is embedding vector of the $j-th$ trajectory.

## Experimental results

### Data description

In this experiment, two datasets are used. One is human mobility trajectory dataset which is called GeoLife dataset. The other is taxi trajectory dataset which is called Porto dataset.

GeoLife trajectory dataset consists of human mobility trajectories. These trajectories are created by 182 people over three years. Location point contained in every trajectory is described by four values, which are timestamp, Latitude, longitude and altitude. This dataset contains 17621 trajectories. And all the trajectories are collected by mobile devices.

Porto dataset consists of taxi trajectories. These taxi trajectories are created by 442 taxies, which are running in Porto, Portugal. These trajectories are also collected by using the mobile devices installed in the taxies. This dataset contains 1.7 million trajectories.

To extract long trajectories contained in these two datasets, location point number of every trajectory should be counted. If a trajectory contains more than 200 location points, it will be identified as a long trajectory. Otherwise, it will be identified as a short trajectory.

### Comparison baseline

Three algorithms are selected to compare with the proposed algorithm. These algorithms are TrajGAT algorithm^32^, traj2vec algorithm^37^ and Traj2SimVec algorithm^38^. Traj2vec algorithm is proposed for performing trajectory clustering. Before executing clustering, traj2vec algorithm is used to transform a trajectory into a vector. During the procedure, firstly, this algorithm uses a slide window to extract features from trajectory. Then a seq2seq auto-encoder method is used to build vector based on extracted features. By using these vectors, similarity value of all the trajectory pairs can be calculated. Traj2SimVec is also a vector construction algorithm. It generates triplet training samples based on training data. Then an encoder is used to map these triplet training samples into similarity space and matching space for getting sample representation vectors. TrajGAT algorithm is mainly proposed for constructing vector for long trajectory. This algorithm has been described in detail before.

### Evaluation method

Currently, the best evaluation methods are self-similarity and cross-similarity comparison^22,39^. These methods try to evaluate the precision of the trajectory similarity computation algorithm based on the calculated *k*-nearest neighbors and real *k*-nearest neighbors. The procedure of these precision evaluation methods are presented in this section.

In this experiment, trajectory dataset is separated into two different sets. One is training set, the other is testing set. Training set is used for training model. Testing set is used for evaluating performance of the model. While executing trajectory similarity computation algorithms based on GeoLife dataset, the testing dataset contains 3000 trajectories. While executing algorithms based on Porto dataset, 10000 trajectories are selected to form testing dataset.

After testing set and training set are obtained, another dataset $N_s$ should be constructed based on testing set. During the construction procedure, *m* sub-trajectories are generated for every trajectory of testing set. All the sub-trajectories are added into set $N_s$. While constructing a sub-trajectory for trajectory *T*, several non-adjacent location points of trajectory *T* should be randomly selected and dropped. Obviously, these *m* sub-trajectories will be treated as the real top-*m* most similar trajectories of trajectory *T*. When a trajectory similarity computation algorithm is running, it will select top-*k* most similar sub-trajectories for every trajectory of testing set from set $N_s$. Based on *k* selected sub-trajectories and *m* generated sub-trajectories of all the trajectories contained in the testing set, evaluation value “HR@10”, “HR@20” and “R10@20” can be obtained. During the procedure, the overlapping part of *k* selected sub-trajectories and *m* generated sub-trajectories is extracted. The proportion of overlapping trajectory number to value *k* should be calculated. This proportion value calculation procedure need to be executed for every trajectory of testing dataset. Finally, the average value of all the proportion values can be computed for every algorithm. Generally, the best algorithm must get the highest average value. If both variable *m* and variable *k* are equal to 10, evaluation value “HR@10” can be obtained from the experiment. If both variable *m* and variable *k* are equal to 20, evaluation value “HR@20” can be obtained. If variable *m* is equal to 20 and variable *k* is equal to 10, evaluation value “R10@20” can be obtained.

### Trajectory similarity computation quality

In this section, the proposed trajectory similarity computation algorithm is compared with TrajGAT algorithm, traj2vec algorithm and Traj2SimVec algorithm. The experimental results are presented in two tables. Table 1 presents the experimental results obtained based on all the long trajectories of GeoLife dataset. Table 2 presents the experimental results obtained based on all the long trajectories of Porto dataset. In these two tables, TrajBOAL represents the proposed algorithm. Cosine similarity is employed to calculate similarity values. The evaluation metrics used in this experiment are “HR@10”, “HR@50” and “R10@50”.

**Table 1:** Experimental Results based on long trajectories extracted from GeoLife Dataset.

	HR@10	HR@20	R10@20
TrajGAT	0.3478	0.4842	0.7235
traj2vec	0.1244	0.1312	0.3567
Traj2SimVec	0.1326	0.1747	0.4175
TrajBOAL	0.421	0.5718	0.8332

**Table 2:** Experimental results based on long trajectories extracted from porto dataset.

	HR@10	HR@20	R10@20
TrajGAT	0.4195	0.5385	0.7691
traj2vec	0.1531	0.1728	0.3852
Traj2SimVec	0.1252	0.1558	0.3569
TrajBOAL	0.5416	0.623	0.8795

From the experimental results, we can find that the proposed algorithm outperforms all the other comparison algorithms. As the proposed algorithm takes both long-term dependence feature and outline feature into consideration and TrajGAT algorithm only takes long-term dependence feature into consideration, it can be concluded that outline feature is important for distinguishing long trajectories. Thus, while calculating similarity value for two long trajectories, the outline feature must be considered. Moreover, it is obvious that TrajGAT algorithm outperforms Traj2SimVec algorithm and traj2vec algorithm. It proves that long-term dependence is also very important while calculating similarity value for long trajectory.

### Trajectory clustering quality

**Figure 8:**
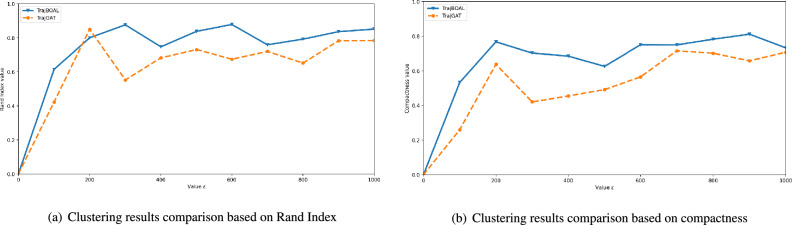
Clustering results obtained based on dataset Porto.

As trajectory similarity computation method is often used in trajectory clustering algorithms, performance of a clustering algorithm using different similarity computation methods are compared in this experiment. The selected clustering algorithm is OPTICS. The similarity computation methods compared in this experiment are TrajGAT algorithm and the proposed algorithm. The clustering result evaluation methods used in this experiment are Rand Index and Compactness. The dataset used for testing clustering algorithm is Porto. The ground truth of clustering results is obtained by using the real similarity value of all the trajectory pairs. As, while the dense neighborhood space radius $\epsilon$ is set to different values, the performance of OPTICS algorithm keeps changing, the clustering algorithm will be tested under different $\epsilon$ value. In (a) and (b) of Fig. 8, the *x*-axis represents different $\epsilon$ value and the *y*-axis represents RI value or Compactness value of the clustering algorithm.

From experimental results presented in (a) and (b) of Fig. 8, we can find that almost all the points on the curve of the proposed algorithm are higher than the points on the curve of TrajGAT algorithm. It means that the clustering algorithm using proposed algorithm outperforms the clustering algorithm using TrajGAT algorithm in both RI value and compactness value. In this situation, we can conclude that the proposed algorithm outperforms TrajGAT algorithm as well. As OPTICS algorithm tries to find clusters based on distance among trajectories, the performance of this clustering algorithm is affected by these distance values. While performing clustering based on trajectory dataset, similarity value of two trajectories will be treated as distance of these two trajectories. So, if two trajectories are very similar, the distance between them will be judged as very close. In this situation, a well-performed trajectory similarity calculation method is very important for improving the performance of OPTICS algorithm. According to this, we can observe the performance of TrajGAT algorithm and the proposed algorithm based on clustering quality of clustering algorithm.

### Trajectory similarity computation quality based on short trajectory set

Based on the experimental results presented above, we can find that the proposed algorithm is better than the compared algorithms while calculating similarity value for long trajectory. In this section, short trajectory dataset is used to test the proposed algorithm. The location point number of every short trajectory is less than 200. Three algorithms are compared with the proposed algorithm, which are Traj2vec, Traj2SimVec and TrajGAT. Three evaluation metrics are used which are “HR@10”, “HR@20” and “R10@20”. Short trajectories are extracted from Porto and Geolife dataset. Experimental results are presented in TableS 3 and 4.

**Table 3:** Experimental results based on short trajectories extracted from GeoLife dataset.

	HR@10	HR@20	R10@20
TrajGAT	0.5319	0.587	0.7811
traj2vec	0.3928	0.4329	0.6753
Traj2SimVec	0.3526	0.408	0.6291
TrajBOAL	0.56	0.5983	0.8141

**Table 4:** Experimental results based on short trajectories extracted from porto dataset.

	HR@10	HR@20	R10@20
TrajGAT	0.5887	0.6124	0.7561
traj2vec	0.5221	0.5536	0.6392
Traj2SimVec	0.3267	0.388	0.5826
TrajBOAL	0.601	0.6212	0.7828

Based on the experimental results, we can find that TrajGAT algorithm performs better than Traj2vec and Traj2SimVec algorithm. It proves that temporal dependence feature is also very important for distinguishing short trajectories. And, at the same time, we can find that the proposed algorithm performs better than TrajGAT algorithm, but “HR@10” value, “HR@20” value and “R10@20” value of these two algorithm are very close. It means that outline feature is not very important for distinguishing short trajectories. This is because, if the trajectory is short, the outline feature may be relatively simple. So the outline feature of many trajectories are very similar. But the proposed algorithm is still better than TrajGAT algorithm. So we can conclude that outline features of some trajectories is totally different from others.

### Influence of embedding dimension

**Figure 9:**
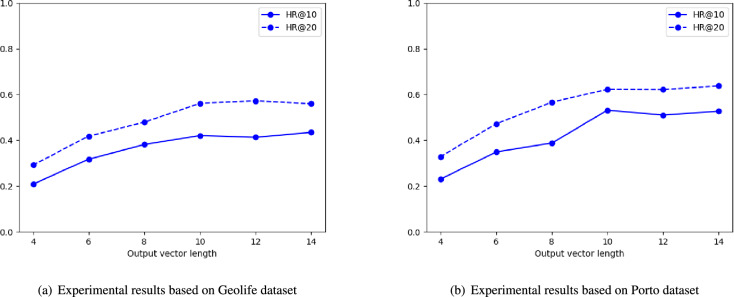
Vector length selection for the output vector of CNN model.

The output vector of CNN model contains outline feature of trajectory. If the output vector is too short, outline feature may be not described clearly by this vector. Thus, to select a suitable length for the output vector, algorithm performance based on different vector length should be tested. In this experiment, the length of output vector will be set to 4, 6, 8, 10, 12, 14. “HR@10” and “HR@20” are used to evaluate the proposed algorithm. Long trajectory set extracted from both Porto dataset and Geolife dataset are used in this experiment. The experimental results are presented in Fig. 9.

Based on the experimental results presented in (a) and (b) of Fig. 9, we can find that, while the length of output vector is less than 10, “HR@10” value and “HR@20” value of the proposed algorithm keep increasing. But, when the length of output vector is greater than 10, the variation of “HR@10” and “HR@20” is small. It means that, if the length of output vector is 10, the outline feature of trajectory can be fully reflected by the output vector. So, while length of output vector is greater than 10, performance of the proposed algorithm remains stable. In this situation, the output vector length of CNN model is set to 10.

### Influence of grid size

**Figure 10:**
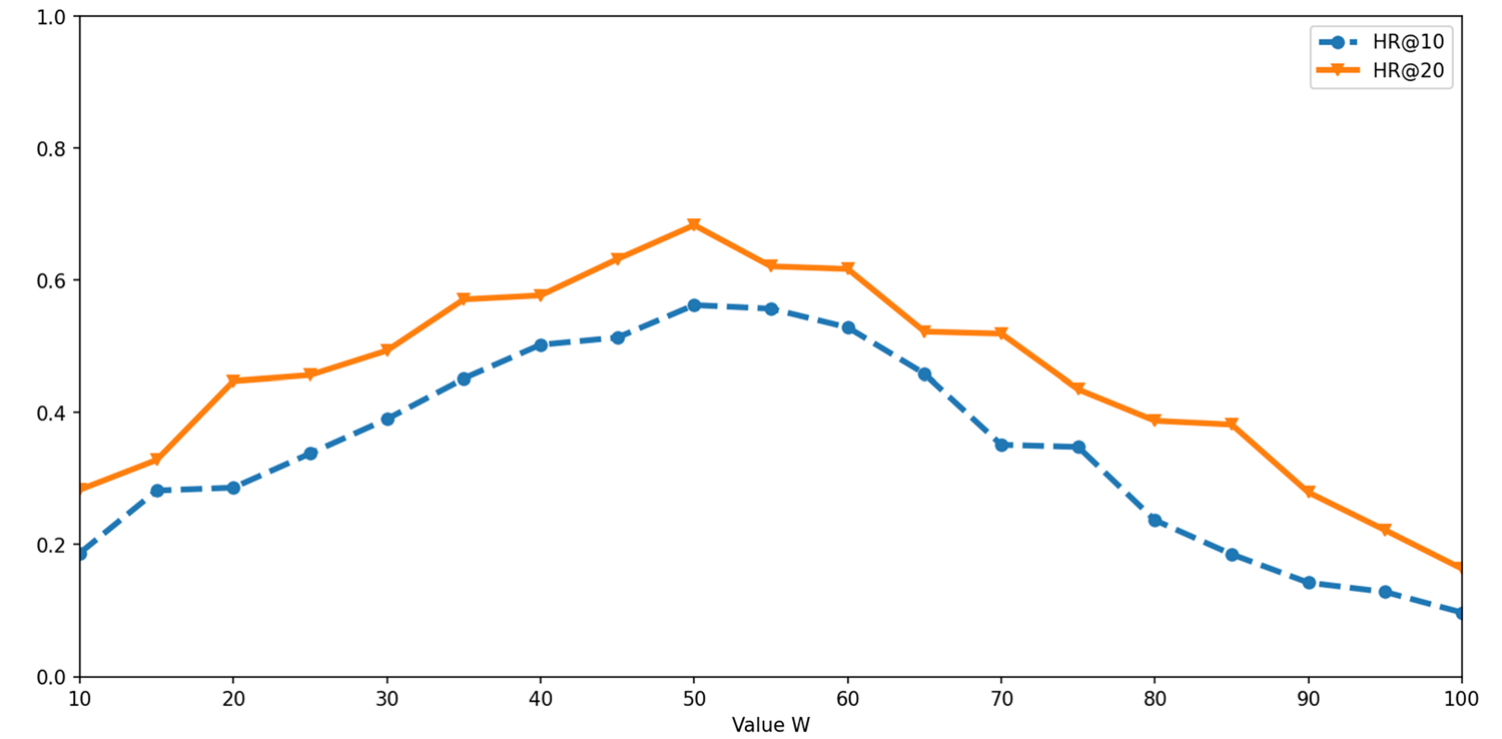
Value “HR@10” and “HR@20” of the proposed algorithm obtained based on different value *W*.

When the proposed algorithm tries to extract outline feature from a trajectory, this trajectory should be projected to a $W \times W$ matrix. Value *W* is a parameter that is decided by users. To select the best *W*, different values should be tested. In this experiment, evaluation metrics “HR@10” and “HR@20” are used to find the best value for parameter *W*. Results of this experiment are presented in Fig. 10. The *x*-axis presents the different values that are assigned to parameter *W*. The *y*-axis presents the values of “HR@10” and “HR@20”.

From the experimental results, we can find that, when parameter *W* is set to 50, “HR@10” and “HR@20” of the proposed algorithm reach the highest value. It means that the best value of parameter *W* is 50. If parameter *W* is too small, resolution ratio of the matrix constructed for every trajectory is bad. So the outline feature can’t be extracted from the matrix precisely. In this situation, performance of the proposed algorithm will be affected. Similarly, if *W* is too large, sparse matrix will be constructed for every trajectory. In this situation, the outline feature of trajectory is distorted. Then, performance of the proposed algorithm will be affected as well.

## Conclusions

Trajectory similarity calculation algorithms are widely used in many kinds of trajectory mining algorithms. It is an important component of trajectory data mining. Nowadays, many trajectory similarity computation algorithms are proposed. But, all these methods don’t take the outline feature of long trajectories into consideration. So, in this paper, a new trajectory similarity calculation algorithm is proposed. This algorithm takes both long-term dependence feature of long trajectory and outline feature of long trajectory into consideration. Thus, GAT-based transformer and CNN model are employed by the proposed algorithm. From experimental results, we can find that the proposed algorithm can cope with long trajectory similarity computation task efficiently. A trajectory is generated by a person who drives a vehicle. Thus the generated trajectory may be affected by the emotion, intention or personal habits of the drivers. In this situation, if all the personal factors are considered while calculating similarity value for trajectories, the performance of similarity calculation algorithm will be improved efficiently. So, in the future, we will try to develop new trajectory similarity computation methods in which the personal factors are considered.

## Data Availability

Dataset GeoLife that support the findings of this study is openly available at https://www.microsoft.com/en-us/download/details.aspx?id=52367. Dataset Porto that support the findings of this study is openly available athttps://tianchi.aliyun.com/dataset/94216.
